# Supplementary material for: Age and learning shapes sound representations in auditory cortex during adolescence
Source: eLife. 2025 Oct 13;14:RP106387. doi: 10.7554/eLife.106387 (PMC12517687; doi:10.7554/eLife.106387)
Supplement: Supplementary file 1. — Linear mixed-effects models of the fixed effects of lick count (until reward or punishment delay), lick latency, cumulative discriminability (d’) (including the interaction effects of lick count and lick latency, lick count and d’, lick latency and d’, and lick latency, lick count and d’) during the minimal number of trials shared between all mice (148 trials; Number of observations = 1098, Fixed effects coefficients = 8, Random effects coefficients = 14, Covariance parameters = 3). Coefficient estimates, STE, T-statistic, degrees of freedom, p-values (adjusted for post-hoc multiple comparisons with Bonferroni method), lower and higher CI are listed in the table. The model includes random effects coefficients per mouse (11 mice in total) and 3 recordings per mouse (see methods, equation 8). Model structure: Lick Count ~ Group * Lick Latency * dprime + (1|Mouse ID) + (1|Recording ID). [file elife-106387-supp1.docx]

| Fixed Effects | Estimate | STE | T-Statistic | DF | P-Value | CI lower | CI upper |
| --- | --- | --- | --- | --- | --- | --- | --- |
| Intercept | 5.441 | 0.2597 | 20.9483 | 1090 | **3.7563e-82** | 4.9314 | 5.9507 |
| Lick Count | -3.4455 | 0.7075 | -4.8697 | 1090 | **1.2827e-06** | -4.8339 | -2.0572 |
| Lick Latency | -0.0086 | 0.0006 | -15.0051 | 1090 | **2.0896e-46** | -0.0098 | -0.0075 |
| d’ | 0.0584 | 0.1102 | 0.5303 | 1090 | 0.5960 | -0.1577 | 0.2746 |
| Count- Latency | 0.0079 | 0.0026 | 3.0258 | 1090 | **0.0076** | 0.0028 | 0.013 |
| Count - d’ | 3.5431 | 1.1445 | 3.0956 | 1090 | **0.006** | 1.2973 | 5.7889 |
| Latency – d’ | -0.0004 | 0.0004 | -0.9635 | 1090 | 0.9999 | -0.0011 | 0.0004 |
| Latency – Count - d’ | -0.0091 | 0.0041 | -2.2524 | 1090 | 0.0735 | -0.0171 | -0.0012 |
