# Supplementary material for: Age and learning shapes sound representations in auditory cortex during adolescence
Source: eLife. 2025 Oct 13;14:RP106387. doi: 10.7554/eLife.106387 (PMC12517687; doi:10.7554/eLife.106387)
Supplement: Supplementary file 2. — Acquired single units, acquired tone-excited units (percentage of tone-excited units relative to total units) in the AUDd, AUDp, AUDv, and TEa of adolescent and adult mice in experts (top) and novice (bottom). [file elife-106387-supp2.docx]

| Expert |  | adolescent |  | adult |  |
| --- | --- | --- | --- | --- | --- |
|  | areas | total | excited | total | excited |
|  | AUDd | 177 | 56 (32%) | 154 | 82 (53%) |
|  | AUDp | 442 | 210 (48%) | 535 | 245 (46%) |
|  | AUDv | 338 | 132 (39%) | 343 | 197 (57%) |
|  | TEa | 188 | 65 (35%) | 235 | 75 (32%) |
| Novice |  | adolescent |  | adult |  |
|  | areas | total. | excited | total | excited |
|  | AUDd | 89 | 22 (25%) | 96 | 17 (18%) |
|  | AUDp | 167 | 31 (19%) | 181 | 49 (27%) |
|  | AUDv | 302 | 47 (16%) | 180 | 65 (36%) |
|  | TEa | 99 | 30 (33%) | 146 | 55 (38%) |
