# Supplementary material for: Age and learning shapes sound representations in auditory cortex during adolescence
Source: eLife. 2025 Oct 13;14:RP106387. doi: 10.7554/eLife.106387 (PMC12517687; doi:10.7554/eLife.106387)
Supplement: Supplementary file 3. — Mean and standard error, mean effect size (robust Cohen’s D), lower and upper Confidence Interval (CI) and p-value (Wilcoxon rank-sum test, adjusted for multiple comparisons with Bonferroni method) of the average baseline FR (Hz), evoked FR (Hz), coefficient of variance of FR, latency to peak of maximal FR (ms), full-width-half maximum of peak FR (ms), minimal latency of first spike (ms), fraction of responsive trials, lifetime sparseness of all adolescent and adult neurons from tone-onset to 50 ms after tone offset across all stimuli in AUDd, AUDp, and AUDv, and TEa (significant p-values are highlighted in bold). [file elife-106387-supp3.docx]

| AUDd | adolescent |  | adult |  |  |  |  |  |
| --- | --- | --- | --- | --- | --- | --- | --- | --- |
| neuronal property | Mean | ± STE | Mean | ± STE | Effect size | lower CI | upper CI | p-value |
| spontaneous FR | 2.4274 | 0.4188 | 2.2271 | 0.3668 | 0.1414 | -0.0111 | 0.2997 | 0.9801 |
| evoked FR | 14.5588 | 1.6046 | 22.1731 | 2.9083 | -0.306 | -0.3798 | -0.1396 | 0.1537 |
| FR coeff. var | 2.4484 | 0.2496 | 1.7209 | 0.1238 | 0.4794 | 0.0573 | 0.3294 | **0.0146** |
| latency to peak | 134.5179 | 8.7109 | 132.061 | 16.1264 | 0.1266 | 0.1826 | 0.5423 | **0.0006** |
| FWHM | 275.7321 | 26.1309 | 245.4634 | 24.4123 | 0.1317 | 0.0409 | 0.2443 | 0.2642 |
| min. latency | 73.5638 | 3.3501 | 48.9258 | 3.1867 | 0.7905 | 0.3529 | 0.6238 | **3.6051E-06** |
| % trials resp. | 0.3809 | 0.0364 | 0.4815 | 0.0328 | -0.2993 | -0.3032 | -0.076 | **0.0422** |
| lifetime sparse. | 0.1744 | 0.0291 | 0.1506 | 0.0183 | 0.0401 | -0.3136 | -0.0548 | 0.7897 |
| AUDp | adolescent |  | adult |  |  |  |  |  |
| neuronal property | Mean | ± STE | Mean | ± STE | Effect size | lower CI | upper CI | p-value |
| spontaneous FR | 3.158 | 0.2654 | 2.7504 | 0.2546 | 0.1752 | -0.0103 | 0.2951 | **0.0115** |
| evoked FR | 24.5512 | 1.6812 | 39.4108 | 2.6007 | -0.432 | -0.4069 | -0.1391 | **5.7054E-05** |
| FR coeff. var | 1.6895 | 0.0872 | 1.4396 | 0.084 | 0.2913 | 0.0522 | 0.3326 | **0.0004** |
| latency to peak | 102.981 | 7.9174 | 50.4 | 3.0841 | 0.7259 | 0.1981 | 0.5378 | **1.0347E-11** |
| FWHM | 241.2571 | 13.7176 | 150.2408 | 12.0679 | 0.4569 | 0.0357 | 0.2445 | **8.7880E-10** |
| min. latency | 47.4148 | 1.8017 | 31.4164 | 1.5054 | 0.6936 | 0.3569 | 0.6333 | **5.605E-12** |
| % trials resp. | 0.517 | 0.0208 | 0.6 | 0.0209 | -0.2354 | -0.2853 | -0.0645 | **0.0026** |
| lifetime sparse. | 0.1459 | 0.0115 | 0.1875 | 0.0118 | -0.2739 | -0.3028 | -0.0485 | **0.0049** |
| AUDv | adolescent |  | adult |  |  |  |  |  |
| neuronal property | Mean | ± STE | Mean | ± STE | Effect size | lower CI | upper CI | p-value |
| spontaneous FR | 2.627 | 0.4173 | 1.9547 | 0.2387 | 0.2687 | -0.0121 | 0.2867 | 0.1084 |
| evoked FR | 21.8639 | 2.8512 | 24.4575 | 1.9949 | -0.1752 | -0.3839 | -0.1324 | 0.4306 |
| FR coeff. var | 1.8877 | 0.1089 | 1.7797 | 0.1009 | 0.1175 | 0.0477 | 0.3122 | 0.0927 |
| latency to peak | 126.1818 | 9.6388 | 135.5939 | 10.1844 | -0.0138 | 0.1951 | 0.5405 | 0.2089 |
| FWHM | 226.5985 | 16.5937 | 241.7817 | 15.1074 | -0.0613 | 0.0334 | 0.2419 | 0.7051 |
| min. latency | 59.9072 | 2.3282 | 51.9611 | 2.1164 | 0.2969 | 0.3359 | 0.6227 | **0.0062** |
| % trials resp. | 0.4461 | 0.0253 | 0.5074 | 0.023 | -0.1811 | -0.2962 | -0.0823 | 0.1396 |
| lifetime sparse. | 0.1593 | 0.0153 | 0.2033 | 0.0127 | -0.3125 | -0.3089 | -0.0527 | **0.0038** |
| TEa | adolescent |  | adult |  |  |  |  |  |
| neuronal property | Mean | ± STE | Mean | ± STE | Effect size | lower CI | upper CI | p-value |
| spontaneous FR | 1.2426 | 0.2315 | 2.1943 | 0.2731 | -0.5523 | -0.0096 | 0.303 | **0.0059** |
| evoked FR | 17.567 | 2.5111 | 16.5377 | 1.7923 | -0.0353 | -0.4066 | -0.1551 | 0.8271 |
| FR coeff. var | 1.9063 | 0.1606 | 2.1603 | 0.2278 | -0.0467 | 0.0585 | 0.3122 | 0.9088 |
| latency to peak | 170.0645 | 18.1797 | 145.8133 | 15.4745 | 0.2663 | 0.2071 | 0.5479 | 0.2447 |
| FWHM | 259.6935 | 25.6747 | 297.2267 | 23.7387 | -0.1590 | 0.038 | 0.2512 | 0.4505 |
| min. latency | 73.5202 | 2.8156 | 59.9417 | 2.8259 | 0.5712 | 0.3687 | 0.6166 | **0.0013** |
| % trials resp. | 0.4412 | 0.0375 | 0.4515 | 0.0347 | -0.0491 | -0.3016 | -0.0768 | 0.909 |
| lifetime sparse. | 0.2156 | 0.0238 | 0.1819 | 0.0206 | 0.2167 | -0.3118 | -0.0521 | 0.1658 |
