# Supplementary material for: Age and learning shapes sound representations in auditory cortex during adolescence
Source: eLife. 2025 Oct 13;14:RP106387. doi: 10.7554/eLife.106387 (PMC12517687; doi:10.7554/eLife.106387)
Supplement: Supplementary file 4. — P-values of Kruskal-Willis Test after Tukey-Kramer correction for multiple comparisons of the average baseline FR (Hz), evoked FR (Hz), coefficient of variance of FR, latency to peak of maximal FR (ms), full-width-half maximum of peak FR (ms), minimal latency of first spike (ms), fraction of responsive trials, lifetime sparseness of all adolescent and adult neurons from tone-onset to 50 ms after tone offset across all stimuli AUDd – AUDp, AUDd – AUDv, AUDd – TEa, AUDp – AUDv, AUDp – Tea, and AUDv –Tea (significant p-values are highlighted in bold). [file elife-106387-supp4.docx]

| adolescent | P-Value** |  |  |  |  |  |
| --- | --- | --- | --- | --- | --- | --- |
| neuronal property | AUDd - AUDp | AUDd - AUDv | AUDd - TEa | AUDp - AUDv | AUDp - TEa | AUDv - TEa |
| spontaneous FR | 0.3379 | 0.9998 | 0.3018 | **0.0793** | **0.0004** | 0.189 |
| evoked FR | **0.0276** | 0.5874 | 0.9983 | 0.2043 | **0.0367** | 0.6856 |
| FR coeff. var | **0.0078** | 0.3519 | 0.5517 | 0.2063 | 0.3573 | 0.9992 |
| latency to peak | **0.0001** | 0.0878 | 0.9894 | **0.0001** | **0.0001** | 0.1681 |
| FWHM | 0.7022 | 0.4202 | 0.998 | 0.8836 | 0.8076 | 0.5188 |
| min. latency | **0.0001** | **0.0263** | 0.9819 | **0.0002** | **0.0001** | **0.0045** |
| % trials resp. | **0.014** | 0.4845 | 0.6757 | 0.1778 | 0.3250 | 0.9992 |
| lifetime sparse. | 0.9998 | 0.9453 | **0.0255** | 0.8245 | **0.0016** | **0.027** |
| adult | P-Value** |  |  |  |  |  |
| neuronal property | AUDd - AUDp | AUDd - AUDv | AUDd - TEa | AUDp - AUDv | AUDp - TEa | AUDv - TEa |
| spontaneous FR | 0.999 | 0.4036 | 0.9081 | 0.2066 | 0.7793 | 0.0985 |
| evoked FR | **0.0004** | 0.9843 | 0.6787 | **0.0001** | **0.0001** | 0.3436 |
| FR coeff. var | **0.0119** | 0.9605 | 0.7967 | **0.0038** | **0.0003** | 0.4040 |
| latency to peak | **0.0001** | 0.6264 | 0.0672 | **0.0001** | **0.0001** | 0.3092 |
| FWHM | **0.0023** | 0.9068 | 0.1614 | **0.0001** | **0.0001** | 0.2782 |
| min. latency | **0.0001** | 0.8888 | **0.0409** | **0.0001** | **0.0001** | 0.0744 |
| % trials resp. | **0.0192** | 0.9373 | 0.9163 | **0.0114** | **0.0018** | 0.5453 |
| lifetime sparse. | 0.2722 | **0.0203** | 0.3678 | 0.4004 | 0.9963 | 0.8102 |
| ** Kruskal Willis Test after Tukey-Kramer correction for multiple comparisons |  |  |  |  |  |  |
