# Supplementary material for: Age and learning shapes sound representations in auditory cortex during adolescence
Source: eLife. 2025 Oct 13;14:RP106387. doi: 10.7554/eLife.106387 (PMC12517687; doi:10.7554/eLife.106387)
Supplement: Supplementary file 6. — P-values of Kruskal-Willis Test after Tukey-Kramer correction for multiple comparisons of the average baseline FR (Hz), evoked FR (Hz), coefficient of variance of FR, latency to peak of maximal FR (ms), full-width-half maximum of peak FR (ms), minimal latency of first spike (ms), fraction of responsive trials, lifetime sparseness of all adolescent and adult neurons from tone-onset to 50 ms after tone offset across all stimuli AUDd – AUDp, AUDd – AUDv, AUDd – TEa, AUDp – AUDv, AUDp – Tea, and AUDv –Tea (significant p-values are highlighted in bold). [file elife-106387-supp6.docx]

| adolescent | P-Value** |  |  |  |  |  |
| --- | --- | --- | --- | --- | --- | --- |
| neuronal property | AUDd - AUDp | AUDd - AUDv | AUDd - TEa | AUDp - AUDv | AUDp - TEa | AUDv - TEa |
| spontaneous FR | 0.3238 | 0.5109 | 0.1699 | 0.9421 | 0.9336 | 0.6709 |
| evoked FR | 0.6254 | 0.9512 | 0.9985 | 0.6134 | **0.0418** | 0.8233 |
| FR coeff. var | 0.2319 | 0.8251 | 0.3252 | 0.3928 | 0.0998 | 0.5767 |
| latency to peak | 0.188 | 0.8251 | 0.9998 | **0.0002** | **0.0449** | 0.7502 |
| FWHM | 0.7308 | 0.3703 | 0.2985 | **0.0009** | **0.0032** | 0.9723 |
| min. latency | 0.3989 | 0.7007 | 0.7987 | **0.0008** | **0.0137** | 0.9999 |
| % trials resp. | 0.2022 | 0.7372 | 0.2238 | 0.4637 | 0.0996 | 0.5149 |
| lifetime sparse. | 0.9927 | 0.9657 | 0.8218 | 0.9944 | 0.0649 | 0.9267 |
|  |  |  |  |  |  |  |
| adult | P-Value** |  |  |  |  |  |
| neuronal property | AUDd - AUDp | AUDd - AUDv | AUDd - TEa | AUDp - AUDv | AUDp - TEa | AUDv - TEa |
| spontaneous FR | 0.4679 | 0.9932 | 0.993 | 0.0533 | 0.3267 | 0.8491 |
| evoked FR | 0.9866 | 0.263 | **0.0141** | **0.0096** | **0.0001** | 0.2526 |
| FR coeff. var | 0.9344 | **0.0143** | **0.0047** | **0.0032** | **0.0006** | 0.9345 |
| latency to peak | 0.8167 | 0.1522 | 0.1139 | 0.3118 | **0.0279** | 0.9939 |
| FWHM | 0.3574 | 0.2036 | 0.0662 | 0.9806 | **0.0413** | 0.848 |
| min. latency | 0.16 | **0.0009** | **0.0068** | 0.0884 | **0.0392** | 0.8875 |
| % trials resp. | 0.9998 | **0.0798** | **0.0174** | **0.0021** | **0.0001** | 0.804 |
| lifetime sparse. | 0.9933 | 0.9319 | 0.9764 | 0.9665 | 0.9972 | 0.9932 |
| ** Kruskal Willis Test after Tukey-Kramer correction for multiple comparisons |  |  |  |  |  |  |
