# Supplementary material for: Age and learning shapes sound representations in auditory cortex during adolescence
Source: eLife. 2025 Oct 13;14:RP106387. doi: 10.7554/eLife.106387 (PMC12517687; doi:10.7554/eLife.106387)
Supplement: Supplementary file 7. — Acquired single units, acquired tone-modulated units, and percentage of modulated units to all acquired units in the AUDd, AUDp, AUDv, and TEa of adolescent and adult mice during passive-listening recordings. [file elife-106387-supp7.docx]

| Group |  |  |  |  |  |
| --- | --- | --- | --- | --- | --- |
| Expert |  | adolescent |  | adult |  |
|  | areas | total | excited | total | excited |
|  | AUDd | 29 | 1 (0.03%) | 64 | 13 (0.2%) |
|  | AUDp | 147 | 44 (0.30%) | 111 | 27 (0.24%) |
|  | AUDv | 137 | 35 (0.26%) | 109 | 25 (0.23%) |
|  | TEa | 35 | 0 (0%) | 124 | 19 (0.15%) |
| Novice |  | adolescent |  | adult |  |
|  | areas | total. | Excited | total | Excited |
|  | AUDd | 89 | 6 (0.07%) | 96 | 8 (0.08%) |
|  | AUDp | 167 | 25 (0.15%) | 181 | 24 (0.13%) |
|  | AUDv | 302 | 44 (0.15%) | 180 | 45 (0.25%) |
|  | TEa | 99 | 17 (0.17%) | 146 | 46 (0.32%) |
