# Supplementary material for: Age and learning shapes sound representations in auditory cortex during adolescence
Source: eLife. 2025 Oct 13;14:RP106387. doi: 10.7554/eLife.106387 (PMC12517687; doi:10.7554/eLife.106387)
Supplement: Supplementary file 8. — Mean and standard error, mean effect size (robust Cohen’s D), lower and upper Confidence Interval (CI) and p-value (Wilcoxon rank-sum test) of the average baseline FR (Hz) (AUDp vs. AUDv: adolescent p = 0.8551; adult p = 0.9711), evoked FR (Hz) (AUDp vs. AUDv: adolescent p = 0.4125; adult p = 0.9954), coefficient of variance of FR (AUDp vs. AUDv: adolescent p = 0.4354; adult p = 0.8800), latency to peak of maximal FR (ms) (AUDp vs. AUDv: adolescent p = 0.5871; adult p = 0.9985), full-width-half maximum of peak FR (ms) (AUDp vs. AUDv: adolescent p = 0.7223; adult p = 0.4628), minimal latency of first spike (ms) (AUDp vs. AUDv: adolescent p = 0.5936; adult p = 0.5669), fraction of responsive trials (AUDp vs. AUDv: adolescent p = 0.3838; adult p = 0.9924), lifetime sparseness (AUDp vs. AUDv: adolescent p = 0.3792; adult p = 0.9341) of all adolescent and adult neurons from tone-onset to 50 ms after tone offset across all stimuli in AUDp, and AUDv (significant p-values are highlighted in bold). [file elife-106387-supp8.docx]

| AUDp | adolescent |  | adult |  |  |  |  |  |
| --- | --- | --- | --- | --- | --- | --- | --- | --- |
| neuronal property | Mean | ± STE | Mean | ± STE | Effect size | lower CI | upper CI | p-value |
| spontaneous FR | 15.0108 | 2.552 | 16.1657 | 2.0109 | -0.1182 | -0.6373 | 0.3627 | 0.8636 |
| evoked FR | 33.9654 | 8.0969 | 40.81 | 4.012 | -0.0807 | -0.5791 | 0.4458 | 0.7709 |
| FR coeff. var | 0.847 | 0.1456 | 0.6278 | 0.1104 | 0.5127 | 0.0149 | 1.0775 | 0.0646 |
| latency to peak | 110.8182 | 8.3515 | 61.963 | 17.0012 | 0.4648 | 0.0048 | 0.9211 | **0.0472** |
| FWHM | 178.3409 | 21.0212 | 68.5185 | 26.5098 | 0.6418 | 0.2081 | 1.0021 | **0.0032** |
| min. latency | 55.5352 | 6.6073 | 48.058 | 4.6893 | 0.242 | -0.2302 | 0.7889 | **0.0389** |
| % trials resp. | 0.6335 | 0.0827 | 0.5972 | 0.5320 | 0.0716 | -0.473 | 0.507 | 0.9951 |
| lifetime sparse. | 0.4198 | 0.0458 | 0.3383 | 0.3580 | 0.2868 | -0.2248 | 0.7884 | 0.1048 |
| AUDv | adolescent |  | adult |  |  |  |  |  |
| neuronal property | Mean | ± STE | Mean | ± STE | Effect size | lower CI | upper CI | p-value |
| spontaneous FR | 18.9295 | 1.8448 | 8.7466 | 3.8163 | 0.47 | -0.0115 | 0.8816 | **0.0174** |
| evoked FR | 46.1598 | 5.3813 | 21.9135 | 8.8535 | 0.6166 | 0.0396 | 1.2171 | **0.0068** |
| FR coeff. var | 1.0532 | 0.232 | 0.9158 | 0.1627 | 0.2143 | -0.3916 | 0.9365 | 0.1785 |
| latency to peak | 109.4857 | 19.6319 | 89.68 | 12.9514 | 0.3197 | -0.3407 | 0.8796 | 0.18106 |
| FWHM | 146.0857 | 30.3333 | 119.2 | 26.5399 | 0.1272 | -0.4042 | 0.7038 | **0.0439** |
| min. latency | 60.4915 | 8.3884 | 49.5169 | 4.3829 | 0.2985 | -0.2216 | 0.8729 | **0.0433** |
| % trials resp. | 0.6946 | 0.0804 | 0.3925 | 0.0545 | 0.7253 | 0.1905 | 1.5257 | **0.0027** |
| lifetime sparse. | 0.3773 | 0.0533 | 0.2565 | 0.0367 | 0.4362 | -0.0431 | 1.1493 | **0.0451** |
