# Supplementary material for: Age and learning shapes sound representations in auditory cortex during adolescence
Source: eLife. 2025 Oct 13;14:RP106387. doi: 10.7554/eLife.106387 (PMC12517687; doi:10.7554/eLife.106387)
Supplement: Supplementary file 9. [file elife-106387-supp9.docx]

| **Group** | **Expert** |  |  | **Novice** |  |  |
| --- | --- | --- | --- | --- | --- | --- |
|  | Mice | Recording | Neurons | Mice | Recording | Neurons |
|  |  |  |  |  |  |  |
| Figure 1 | 15 (adolescent)  15 (adult) | none | none | none | none | none |
| Figure 2 | 15 (adolescent)  15 (adult) | none | none | none | none | none |
| Figure 3 | 5 (adolescent)  6 (adult) | none | none | none | none | none |
| Figure S3-2 | 4 (GtACR2)  3 (dTomato) | none | none | none | none | none |
| Figure 4 | 5 (adolescent)  6 (adult) | 13 (adolescent)  14 (adult) | 1145 (adolescent)  1267 (adult) | none | none | none |
| Figure 5 | 5 (adolescent)  6 (adult) | 13 (adolescent)  14 (adult) | 1145 (adolescent)  1267 (adult | none | none | none |
| Figure 6 | 5 (adolescent)  6 (adult) | 13 (adolescent)  14 (adult) | 1145 (adolescent)  1267 (adult) | 3 (adolescent)  3 (adult) | 6 (adolescent)  6 (adult) | 657 (adolescent)  603 (adult) |
| Figure 7 | 4 (adolescent)  4 (adult) | 4 (adolescent)  4 (adult) | 348 (adolescent)  408 (adult) | 3 (adolescent)  3 (adult) | 6 (adolescent)  6 (adult) | 557 (adolescent)  503 (adult) |
